# Qualitative and quantitative endothelium changes after cataract surgery: ultrasound phacoemulsification vs. nanolaser technique

**DOI:** 10.3389/fmed.2023.1097404

**Published:** 2023-09-21

**Authors:** Walid Zbiba, Malek Kharrat, Sana Sayadi, Zeineb Kallel, Ghassen Marzouk

**Affiliations:** Department of Ophthalmology, Mohamed Taher Maamouri Hospital, Faculty of Medicine Tunis El Manar, Nabeul, Tunisia

**Keywords:** cataract surgery, phacoemulsification, nanosecond laser, endothelial cells density, endothelial cells morphology

## Abstract

**Purpose:**

The aim of this study was to evaluate corneal endothelial cell density and morphology, central corneal thickness, and best visual acuity using ultrasound (US) phacoemulsification or nanosecond laser technique.

**Setting:**

Department of ophthalmology, Nabeul, Tunisia.

**Design:**

Prospective cohort study.

**Methods:**

This study included eyes with nuclear cataracts with a density grade of 1, 2, 3, or 4 according to LOCS III, divided into two groups; group 1 had conventional US, and group 2 had nanosecond laser. The endothelial cell density (ECD), coefficient of variation (CoV) in cell size, percentage of hexagonal cells, central corneal thickness (CCT) and best visual acuity (VA) were evaluated during 24 months.

**Results:**

Seventy-four eyes had uneventful surgery, 40 in group 1, 34 in group 2. Three procedures in group 2 required conversion to standard phacoemulsification. The mean ECD decreased from 2616.4 ± 194.6 cells/mm^2^ in group 1 preoperatively to 2088.4 ± 229.9 after 2 years. In group 2, it decreased from 2611.8 ± 186.5 cells/mm^2^ to 2276.4 ± 163.8 after 2 years. The change was statistically significant in both groups. The decline of the mean ECD in group 2 was significantly less important than in group 1 (*p* = <10^−2^). The mean percentage of hexagonal cells was 45.18 ± 4.9 preoperatively and 43.5% ± 6.6 after 2 years in group 1. In group 2, it remained almost stable with 45.6 ± 5.1 and 45.4% ± 6.6 preoperatively and after 2 years, respectively. Preoperatively, the mean CoV was 0.39 ± 0.037 in group 1 and 0.38 ± 0.04 in group 2. After 2 years, it was 0.38 ± 0.04 and 0.37 ± 0.038 in group 1 and group 2, respectively. The changes of the mean CoV and the mean percentage of hexagonal cells were significant in both groups, but the difference between the groups was significant only during the six first months postoperatively. In preoperative, the mean corneal central thickness was 509.7 ± 19.5 in group 1 and 510.3 ± 20.4 in group 2. In both groups, the mean corneal thickness increased on D1 postoperatively to 550.9 in group 1, and 528.2 in group 2. The mean corneal thickness decreased more rapidly after 1 week in group 2, to find the initial values. Visual acuity improved from 0.76 Log Mar ± 0.5 at enrolment to 0.45 Log Mar ± 0.2, and 0.033 Log Mar ± 0.086 in group 1 at 1 day post-operative and after 24 months, respectively and from 0.58 Log Mar ± 0.28 to 0.2 Log Mar ± 0.09 and 0.035 Log Mar ± 0.083, respectively in group 2. There was no significant difference in VA at each follow-up between groups except for day 1.

**Conclusion:**

Our study showed lower corneal tissue trauma, and lower endothelial cell loss in the laser cataract surgery compared to phacoemulsification.

**Clinical trial registration**: (https://classic.clinicaltrials.gov/ct2/show/NCT05886283), identifier NCT05886283.

## Introduction

Cataracts are the leading cause of curable blindness worldwide (1). The only treatment available for cataracts is surgery. Currently, the most widely practiced eye surgery worldwide is ultrasound phacoemulsification (US PHACO). Despite gradual improvements and refinements in US PHACO over time, there is still an unavoidable decrease in endothelial cell density (ECD) after any cataract surgery (2–5). Significant loss in cell morphology could potentially compromise corneal transparency and affect functional outcomes (5–10).

Laser technology is now more commonly used in ophthalmic surgery, including excimer laser, Femtolaser, and nanosecond lasers. Cataract laser surgery is gaining momentum. Initially introduced in cataract surgery by Dodick in 1991 (11, 12), this technique appears to be safer and causes less damage to the corneal endothelium, thereby allowing for better visual rehabilitation.

The objective of this study was to compare corneal endothelial cell density and morphology, central corneal thickness, and best-corrected visual acuity (BCVA) between two groups of patients who underwent either US PHACO or nanosecond laser technique (NL PHACO).

## Methods

A prospective randomized uncontrolled cohort study was conducted at the Department of Ophthalmology of Mohamed Taher Maamouri Hospital (MTMH) from March 2017 to May 2020, with approval from the MTMH ethics committee. Informed consent was obtained from all patients, and the study adhered to the principles outlined in the Declaration of Helsinki.

The inclusion criteria involved patients over 50 years old with senile cataract. A comprehensive ocular examination was performed, including visual acuity assessment, clinical corneal evaluation, intraocular pressure (IOP) measurement, and grading of nuclear hardness based on the Lens Opacity Classification System III (LOCS III), which is commonly used in most studies (13, 14). Only the nuclear component of the cataract (NO1 to NO4) was considered for evaluation.

The central corneal endothelium was examined using a non-contact specular microscope (TOMEY CORP EM-4000). Patients with preoperative endothelial cell count (ECC) less than 1,500 cells/mm^2^ and those with pathological alterations in the anterior segment, such as corneal opacities, cornea Guttata, uveitis, pseudoexfoliative syndrome, glaucoma, high myopia axial length (≥26 mm), or chronic pathologies that may affect the corneal endothelium (e.g., diabetes), were excluded. Patients requiring a conversion from NL PHACO to US PHACO or experiencing complications such as capsular rent and vitreous loss were also excluded. All surgeries were performed by a single experienced surgeon (WZ).

The patients were randomly divided into two groups using a block randomization. Group 1 underwent US PHACO using the phaco-chop technique with the Stellaris PC system (Bausch + Lomb^®^), while group 2 underwent NL PHACO using the Cetus A.R.C. Laser system^®^. The Cetus A.R.C. Laser system consists of a base that generates a 4 to 5 ns pulsed Nd:YAG laser with a wavelength of 1,064 nm, a pulse frequency of up to 10 Hz, and an optic fiber that transmits the laser pulses to a disposable single-use coaxial handpiece. The probe used had a total diameter of 720 μm, with a 320 μm quartz optical fiber in the center that directed the laser pulse towards a titanium plate in the phaco probe. The individual pulse energy ranged from 30% to 50% of maximum power, and the pulse frequency was 1–2 Hz. The base unit was connected to the same phaco aspiration/irrigation system and controlled by its pedal.

Both groups underwent surgery starting with a clear corneal incision of 2.2 mm at 9 o’clock and a port incision of 1 to 1.5 mm at 2 o’clock, using the same knife. A continuous circular capsulorhexis (CCC) of 6 mm was performed under the ophthalmic viscosurgical device DuoVisc^®^ (3% sodium hyaluronate, 4% chondroitin sulfate, Alcon). Hydrodissection and phacoemulsification followed.

**Figure 1:**
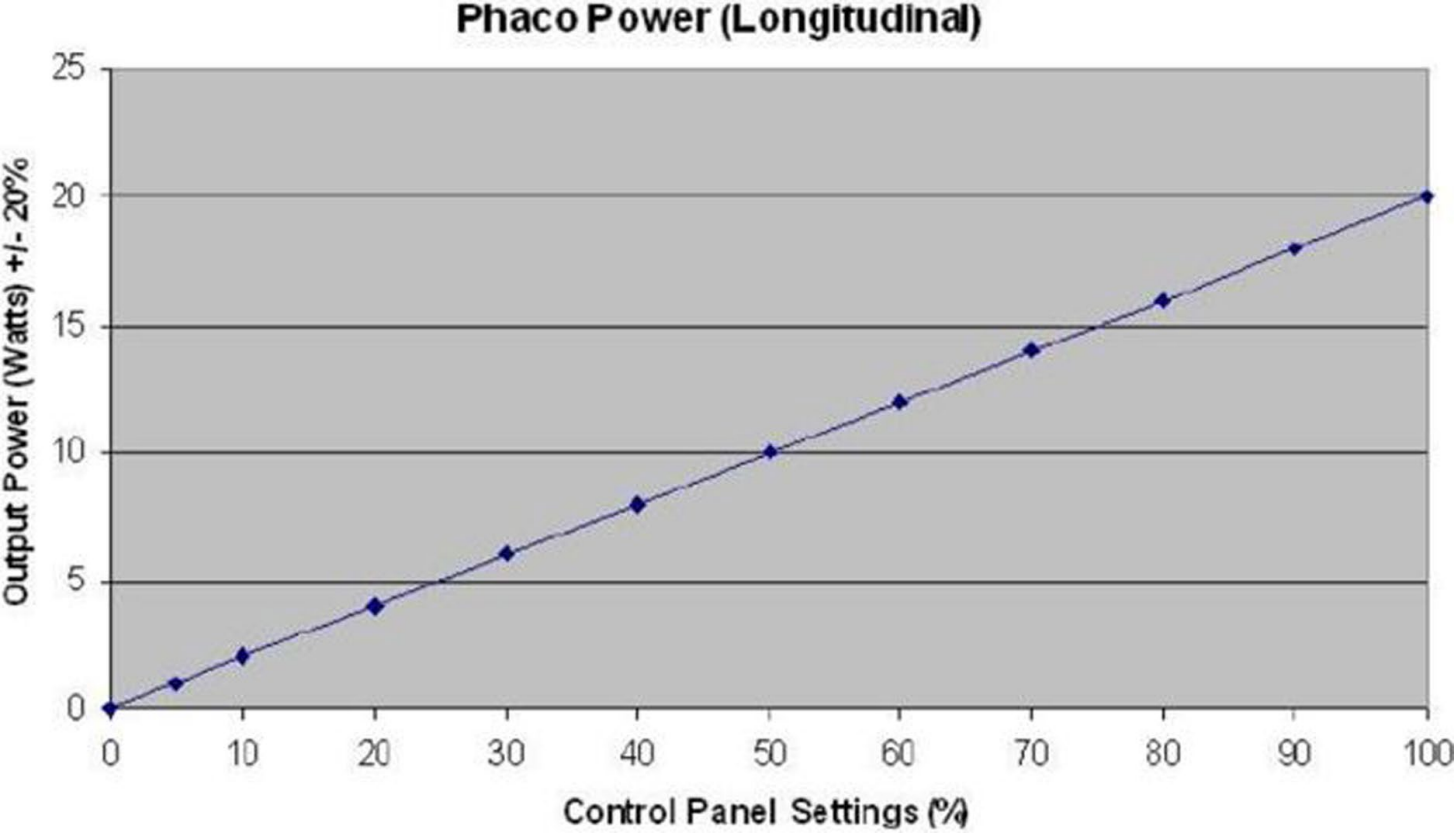
Conversion curve of energy into watts. Phaco, phacoemulsifucation.

In group 1 (horizontal phaco chop), after cortex aspiration, the nucleus was held with the phaco tip at high vacuum. The phaco chopper was then introduced from the side port incision to engage the lower edge of the CCC. It was drawn towards the phaco tip to cleave the nucleus through manual separation between the two instruments. This process continued for the two nuclear halves, and the fragments were aspirated with phaco power. The standard parameters used during phacoemulsification were a vacuum level of 500 mmHg, pressurized irrigation of 90 mmHg, and 40% of phaco power. Energy was expressed as a percentage on the PHACO machine and initially converted into Watts based on a specific curve (Figure 1), and then into joules using the formula: Energy (joules) = Power (watts) × Time (seconds).

In group 2, the cortex and epinucleus were aspirated. The nucleus was fragmented using shockwaves emitted from the phaco tip and then aspirated. The phaco chopper was used to accelerate the mechanical fragmentation of the nucleus and minimize energy dissipation. The energy used was automatically calculated by the ARC Laser machine and displayed on the screen.

In both groups, after bimanual infusion/aspiration for cortex removal and Visco expansion of the capsular bag, a hydrophobic single-piece AcrySof acrylic IOL (SA60AT, Alcon) was implanted. The duration of the entire procedure (in minutes) was recorded.

Uncorrected visual acuity (UVA) was assessed at 1 day postoperative, and best corrected visual acuity (BCVA) was measured at 1 week, 1, 3, 6, and 24 months after surgery. Corneal edema and anterior chamber (AC) reaction were also recorded. A visual outcome of 0.5 logarithm of the minimal angle of resolution (logMAR) without correction was considered a successful result, following the definition provided by the World Health Organization (1, 15, 16). The specular microscope examination was performed at 1 week, 1, 3, 6, and 24 months postoperatively, reporting endothelial cell density (EDC) (cells/mm^2^), the percentage of hexagonal cells, coefficient of variation (CoV) in cell size, and central corneal thickness (CCT) at each follow-up visit.

All data were analyzed using SPSS software (version 21.0; SPSS, Inc., Chicago, IL, United States). BCVA data were converted into logMAR for statistical analysis. Quantitative variables were presented as medians, means, and standard deviations. Student’s *t*-test for independent series was used, and Pearson’s correlation coefficient was calculated to determine statistical relationships or associations between variables. A *p*-value of <0.05 was considered statistically significant.

## Results

Our study included 74 eyes from 57 patients who underwent uneventful phacoemulsification: 40 patients in group 1 and 34 patients in group 2. The demographic and preoperative data of our study population are summarized in Table 1. The two groups were statistically comparable, with the only comorbidity observed being hypertension in 28.4% of patients (11 patients).

**Table 1:** Summary of the preoperative data for the two groups.

		US PHACO (*n* = 40)	Laser PHACO (*n* = 34)	*p*
Age		68.2 ± 6.7	66.38 ± 7.7	0.411^a^
Best corrected visual acuity (LogMar)		0.76 ± 0.5	0.58 ± 0.28	0.131^a^
Astigmatisme (D)		0.69 ± 0.41	1.01 ± 0.76	0.58^a^
Mean Cataract density (LOCSIII)		2.45	2.47	0.101^a^
Cataract density (LOCS III) (*n*)	NO1	5	4	
	NO2	21	18	
	NO3	12	12	
	NO4	2	0	
Axial length (mm)		23.19	23.3	0.534^a^
IOL power (D)		21.55 ± 1.41	21.66 ± 1.7	0.75^a^
ECD (c/mm^2^)		2616.4 ± 194.6	2611.8 ± 186.5	0.918^a^
Hexagonality (%)		45.18 ± 4.9	45.6 ± 5.1	0.725^a^
CoV		0.39 ± 0.037	0.8 ± 0.04	0.249^a^
CCT (μm)		509.7 ± 19.5	510.3 ± 20.4	0.894^a^

US, ultrasound; PHACO, phacoemulsification; D, diopter; *n*, number of patients; IOL, intraocular lens; ECD, endothelial cell density; CoV, coefficient of variation; CCT, central corneal thickness.

a Student *t*-test.

### Perioperative data

Mean intervention time was 10.1 min ± 2.2 in group 1 and 11.97 min ± 2.5 in group 2 (*p* = 0.001). The energy dissipated during the operation was 245.2 J ± 193.1 in group1 and 2.16 ± 0.8 J in group 2 (*p* < 10^−2^). The mean intervention time and dissipated energy during the operation depending on cataract density were noted in “Table 2.” In three patients from group 2 with NO4 cataracts, surgery was converted to US PHACO, and patients were excluded from the study. We noted one case of posterior capsule rupture in group 2, that resulted from direct contact between the laser probe and the capsule.

**Table 2:** Mean intervention time and dissipated energy during the operation depending on cataract density.

	Energy (joules)			Surgery time (second)		
	Group 1	Group 2	*p*	Group 1	Group 2	*p*
NO1	79.2 ± 48.6	1.5 ± 0.4	<10^**−**2*^	50 ± 25.5	127.5 ± 37.7	<0.05^*^
NO2	175.4 ± 85.9	2 ± 0.6	<10^**−**2*^	81.5 ± 34.7	137.9 ± 69.6	<0.05^*^
NO3	342.4 ± 154	2.7 ± 0.9	<10^**−**2*^	135.6 ± 58.5	214.4 ± 86.6	<0.05^*^

NO indicates nuclear density according to LOCSIII classification. ^*^Student *t*-test.

### Post-operative data

On the first day after surgery, corneal edema was observed in 72.5% of the US group (17.5% diffuse edema, 50% sectoral edema, and 5% disciform), and in 52.9% of cases in the laser group (100% sectoral edema). The difference in the incidence of corneal edema between the two groups was statistically significant (*p* = 0.02).

Two cases of Irvine Gass syndrome were observed only in group 1. No cases of endophthalmitis, bullous keratopathy, or retinal detachment were reported during the follow-up period.

The mean endothelial cell density (ECD) decreased from 2,616 cells/mm^2^ ± 194.6 (SD) in group 1 preoperatively to 2088.4 ± 229.9 (SD) after 24 months, and from 2611.8 cells/mm^2^ ± 186.5 (SD) to 2276.4 ± 163.8 (SD) in group 2. The detailed evolution of ECD in the two groups is presented in Table 3. The change in ECD before and after surgery was statistically significant in both groups (*p* < 10^−2^). The decline in mean ECD in group 2 was statistically less significant than in group 1 and persisted throughout the two-year follow-up (*p* < 10^−2^).

**Table 3:** Evolution of ECD in the two groups.

	ECD (cell/mm^2^)		
	Group 1	Group 2	*p*
Preoperative	2,616 ± 194	2,611 ± 186	<10^**−**2*^
Day 7	2,226 ± 234	2,355 ± 178	<10^**−**2*^
1 month	2,160 ± 235	2,314 ± 166	<10^**−**2*^
3 months	2,126 ± 236	2,301 ± 186	<10^**−**2*^
6 months	2,103 ± 230	2,286 ± 166	<10^**−**2*^
24 months	2,088 ± 229	2,276 ± 163	<10^**−**2*^

ECD, endothelial cell loss. ^*^Student *t*-test.

The most significant endothelial cell loss (ECL) was observed in the first week postoperatively in both groups. ECL continued until the two-year follow-up but at a slower rate over time. The difference in ECL between the two groups was statistically significant (*p* < 10^−2^) only during the immediate postoperative period (1 month after surgery).

We evaluated the percentage of ECL at 1 week postoperatively based on cataract density and the groups (see Table 4).

**Table 4:** Percentage of endothelial cellular loss at 1 week postoperatively in function of cataract density in the two groups.

	ECD %		
	Group 1	Group 2	*p*
NO1	9.1 ± 3.5	8.4 ± 6.2	0.856^*^
NO2	13.2 ± 3.9	9.4 ± 5.9	0.022^*^
NO3	19.7 ± 3.4	10.4 ± 6.4	<10^−2*^
NO4	19.9 ± 4		—

ECD, endothelial cell density. ^*^Student *t*-test.

**Figure 2:**
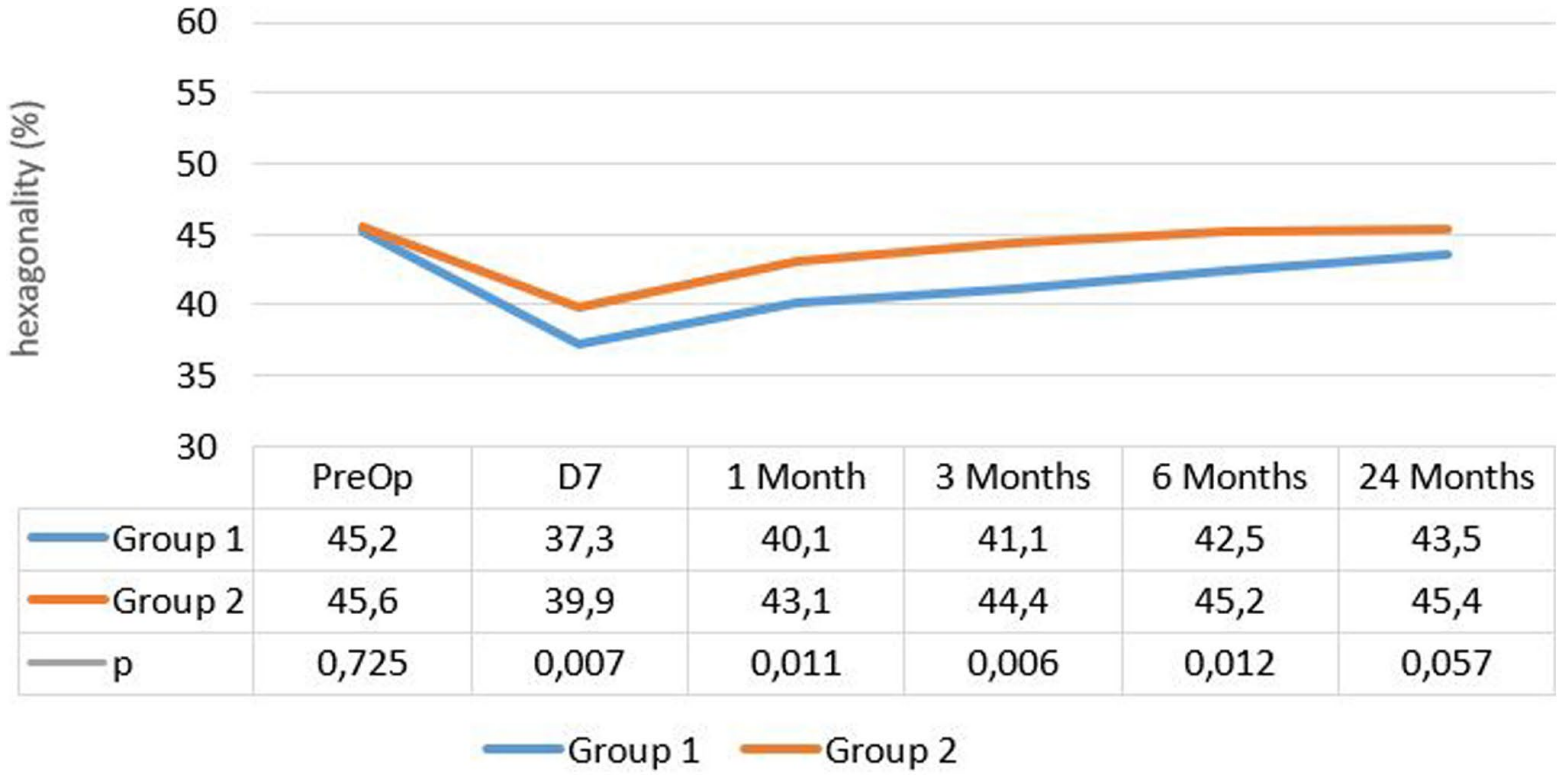
Evolution of hexagonal cells percentage in both groups.

The mean percentage of hexagonal cells was 45.18% ± 4.9 at enrollment and 43.5% ± 6.6 after 24 months in group 1, while it was 45.6% ± 5.1 at enrollment and 45.4% ± 6.6 after 24 months in group 2, without a significant difference in either group (*p* = 0.114). However, a significant decrease in hexagonality was observed at the 1 week follow-up in each group (*p* < 10^−2^) (see Figure 2).

**Figure 3:**
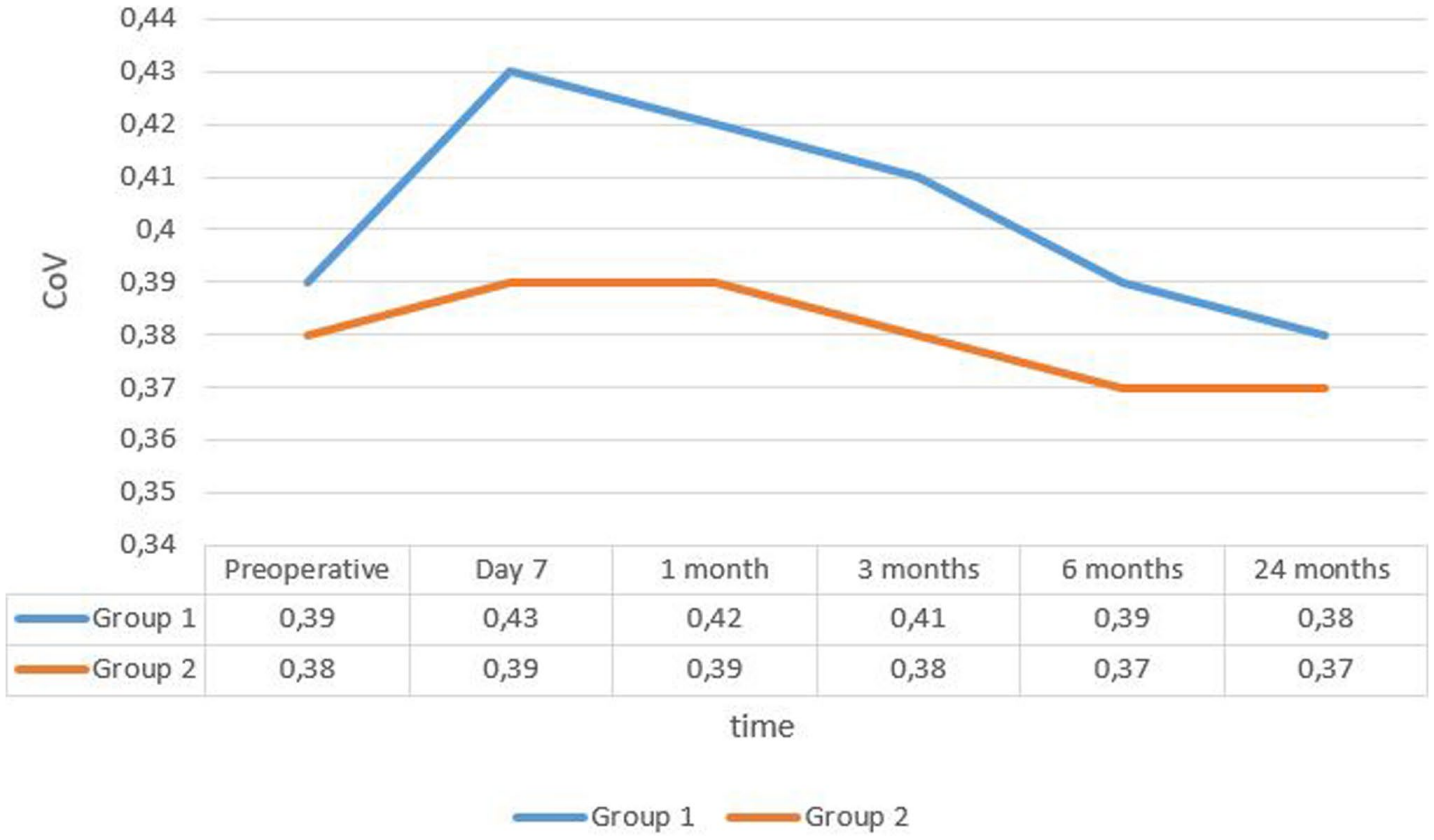
Evolution rate of mean covariance in the two groups. CoV, coefficient of variance.

The mean coefficient of variation (CoV) in group 1 was 0.39 ± 0.037 at enrollment and 0.38 ± 0.04 after 24 months. In group 2, it was 0.38 ± 0.04 and 0.37 ± 0.038, respectively (see Figure 3).

The change in mean CoV and mean percentage of hexagonal cells was significant in both groups only in the first month (*p* < 0.05), but the difference between the groups was significant during the first 6 months postoperatively.

**Figure 4:**
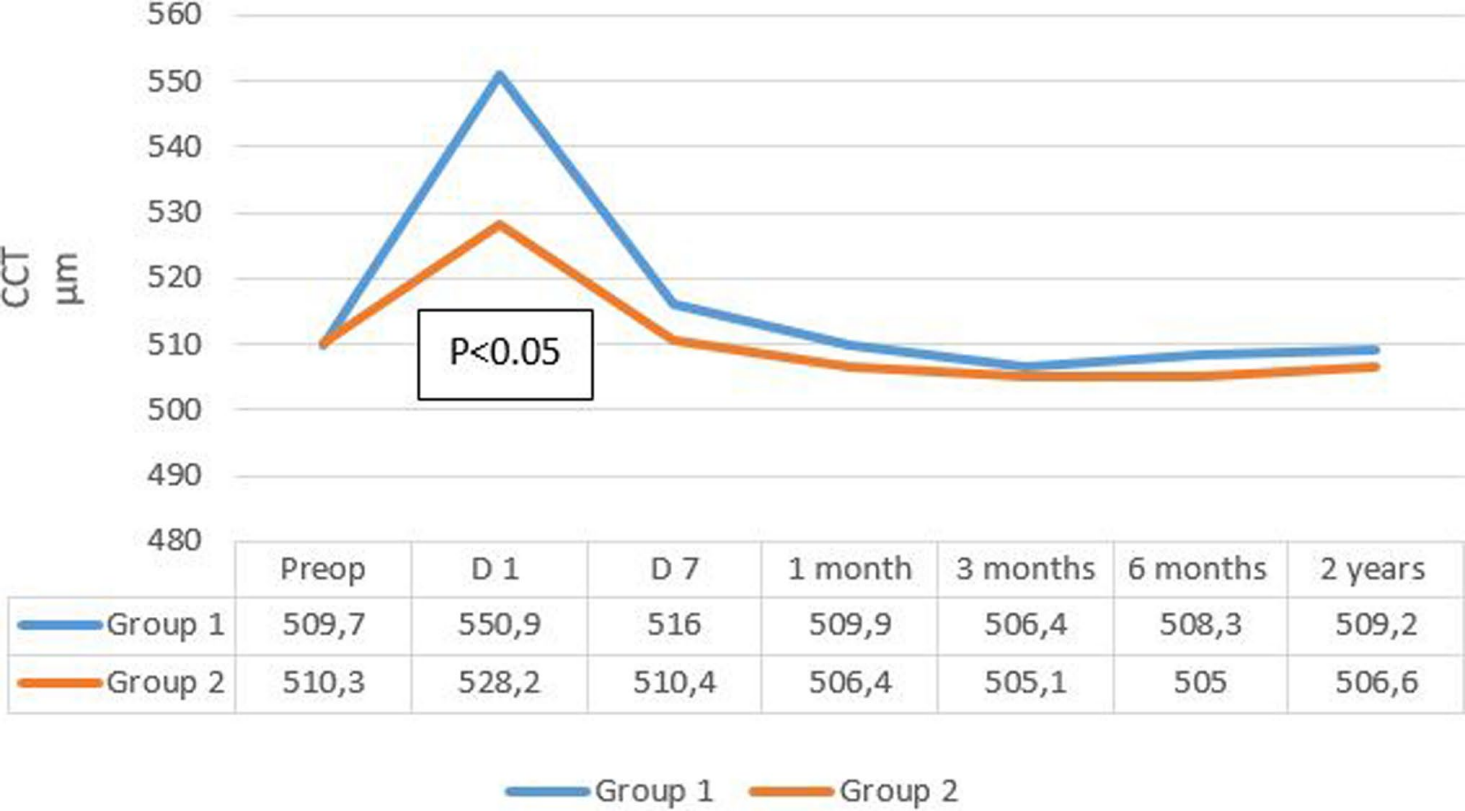
Evolution rate of mean CCT in the two groups. CCT, central corneal thickness.

The evolution of mean central corneal thickness (CCT) in the two groups is summarized in Figure 4. Mean CCT decreased more rapidly after 7 days in group 2, reaching its initial values, while in group 1, the decline was less pronounced, and the CCT remained higher compared to baseline values.

CCT measurements at 1 day postoperative showed a significant increase in both groups regardless of the cataract density (*p* < 0.05). It decreased at the 1 week follow-up and returned to baseline values in group 2 but not in group 1.

Furthermore, for grade 4 cataracts in group 1, a decrease in CCT was observed on day 7 postoperative, but without returning to preoperative values, and without a significant difference compared to the values observed on day 1 postoperative (*p* = 0.156).

**Figure 5:**
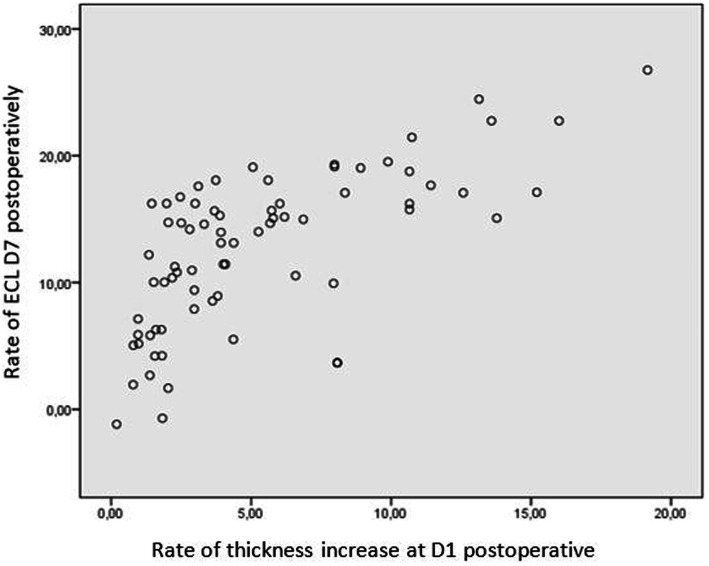
Correlation between endothelial cell loss and CCT at day 1 postoperative. CCT, central corneal thickness.

A significant correlation was observed between endothelial cell loss and CCT on day 1 postoperative in both groups. A decrease in CCT of less than 5% from the preoperative values corresponded to an endothelial cell loss of 9.2 ± 5.1%, while an increase of more than 15% correlated with a loss of 20.9 ± 47%, respectively (Figure 5).

### Functional results

Visual acuity improved from 0.76 LogMAR ± 0.5 at enrollment to 0.45 LogMAR ± 0.2 and 0.033 LogMAR ± 0.086 in group 1 at 1 day postoperative and after 24 months, respectively. In group 2, visual acuity improved from 0.58 LogMAR ± 0.28 to 0.2 LogMAR ± 0.09 and 0.035 LogMAR ± 0.083, respectively (Figure 5). There was no significant difference in visual acuity at each follow-up between the groups, except for day 1.

## Discussion

Phacoemulsification is considered the gold standard in cataract surgery. While endothelial cell damage remains inevitable, studies have shown that new surgical techniques may be safer for the corneal endothelium despite manipulation in the anterior chamber (15).

In our study, we present the results of the first cohort in Africa treated with nanolaser, with a prospective 2 years follow-up. The surgeries were performed by the same experienced surgeon to minimize bias and ensure consistency.

Nanolaser photophacolysis is a recently developed surgical technique that appears to cause less damage to the corneal endothelium (16). In this prospective randomized study, we compared the impact of US phacoemulsification and nanolaser on endothelial structure, corneal edema, central corneal thickness (CCT), and evaluated the refractive outcomes.

The two groups were well-matched in terms of preoperative parameters, including age, best-corrected visual acuity (BCVA), and nucleus density. Preoperative specular microscopy data [endothelial cell density (ECD), coefficient of variation (CoV), and hexagonality] were comparable between the two groups and fell within the normal range reported in the literature (17, 18), except for a slightly lower hexagonality rate. The ECD in our cohort was thinner than reported in other studies, possibly due to anatomical variations among regions (19).

Two papers (12, 20) stated posterior capsule ruptures as a possible complication during the NL PHACO. We noted one case of this complication in the second group. The data in our study (only one case of posterior capsule rent) is insufficient to establish a complication rate. A larger study can provide a more proximate percentage.

Corneal edema was the earliest complication observed in both groups. It was significantly more prevalent in group 1 compared to group 2 and generally decreased starting from postoperative day 7.

Although the nanolaser group had a longer mean intervention time, the total amount of energy emitted from the nanosecond laser tip was significantly lower than that from the US probe, which aligns with the findings of Mastropasqua et al. (21) regarding laser cataract surgery. Few cases of bullous keratopathy related to the laser technique were reported in initial studies (17, 20), but none occurred in our cohort. Corneal edema appears to be directly related to endothelial cell loss resulting from corneal injuries during surgery.

The percentage of cell loss showed a slight decrease at 3 months postoperatively in both groups, with no significant difference compared to the first month. The time required for ECD stabilization after surgery remains a topic of controversy (22). Most authors suggest that endothelial cell loss stabilizes between 3 and 6 months postoperatively (23, 24). However, in our study, the ECD continued to decrease over 2 years, possibly due to physiological loss.

At the last follow-up, the mean loss of endothelial cells was 20.2% in the US phacoemulsification group, while it was only 12.7% in the nanolaser phacoemulsification group. This difference was statistically significant. Tanev et al. (16) reported similar results after a 2 years follow-up, while Vergés et al. (17) found that US phacoemulsification caused less endothelial damage than laser mediated surgery, particularly in cases of dense cataracts.

Cataract density is considered the most important factor in determining endothelial cell loss after cataract surgery (2, 25, 26). Regardless of the technique used, higher cataract density is associated with greater endothelial cell loss. In our cohort, there were no significant differences in endothelial damage observed in grade 1 cataracts. However, for grade 2 and 3 cataracts, there was a significant difference between the two groups.

Mishima (7) confirmed that the analysis of cell shape and form is a more sensitive indicator of endothelial damage than ECD. In fact, endothelial cell loss is directly related to surgical trauma, while cell form and pattern play a more significant role in the regeneration process after surgery.

Regarding morphometric parameters, significant changes in CoV and hexagonality occurred in both groups after surgery, with significant modifications observed at 1 week postoperatively. The values returned to the normal range within 3 months from the surgery date. One week after surgery, the rate of hexagonal cells dropped in both groups, with a more pronounced decline in group 1, while the coefficient of variation increased in both groups, particularly in group 1. At the last follow-up, group 2 exhibited lower CoV and higher hexagonality rates.

Tanev et al. (16) reached the same findings, concluding that the morphologic status stabilizes within 6 months. However, Atas et al. found no variation in hexagonality or coefficient of variation before and after surgery. This can be explained by the delay of the first post-surgery check performed at 1 month, which did not evaluate the changes in the immediate postoperative period. According to our study and the literature, these immediate postoperative variations are considered the most significant (27).

Furthermore, corneal edema arises from endothelial pump dysfunction and can be objectively evaluated through variations in corneal thickness (28). In our cohort, central corneal thickness (CCT) increased by 6% from baseline at 1 day postoperative and returned to normal values within 7 days. The difference between the two groups was statistically significant, indicating a higher incidence of postoperative edema in group 1. This increase in CCT also appears to be associated with cataract density, as observed by Huetz et al. (15) and Vergès et al. (17), who found significant changes primarily in grade 3 cataracts or higher.

It is worth noting the established linear correlation between CCT and endothelial cell density (ECD) (29, 30). Perone et al. (31) demonstrated that when CCT increased by more than 15%, endothelial cell loss was approximately 15%, whereas an increase of less than 5% in CCT corresponded to less than 5% loss of endothelial cells. Our findings align with previous studies, emphasizing the potential value of pre-and postoperative CCT evaluation as a predictive tool for surgical-induced endothelial damage.

Immediate postoperative visual acuity was significantly better in group 2 compared to group 1, attributed to a lower incidence of postoperative corneal edema. Previous studies have not reported significant differences in visual acuity between nanolaser and classic phaco chop techniques. At the last follow up, best-corrected visual acuity (BCVA) was comparable between the two groups, indicating equivalent successful outcomes for both techniques. Nevertheless, it can be assessed that laser surgery enables earlier functional rehabilitation and satisfactory refractive outcomes. In clinical practice, considering the reduced endothelial damage associated with nanolaser, it presents an intriguing perspective for patients with corneal dystrophies or low endothelial cell density. Furthermore, for individuals requiring swift visual recovery for occupational purposes, such as drivers, architects, or frequent travelers, nano laser may offer a more suitable alternative to classic phaco.

In a recent study, Sauder reported the feasibility of extracting grade 4 and 5 cataracts using newly designed probes with a larger luminal cross sectional area (29).

One limitation of our study was the exclusion of patients with grade 4 cataract density in group 2. This decision was based on the longer operating time and the requirement for a second nanosecond laser probe tip during the same surgery, which significantly increased the intervention cost. Another limitation was the inclusion of both eyes of 15 patients, which introduces the potential for selection bias due to the similarity in corneal parameters between the eyes.

## Conclusion

Our study demonstrated that nanolaser causes less corneal tissue trauma and reduces endothelial cell loss compared to phacoemulsification in cataracts of grade 1, 2, and 3 according to the Lens Opacities Classification System III (LOCSIII). Notably, nanolaser enables earlier visual rehabilitation, particularly in grade 1 cataracts. However, for grade 4 cataracts, further investigations employing suitable nanolaser probes are required to better evaluate the corneal endothelial changes following surgery.

## Data availability statement

The original contributions presented in the study are included in the article/supplementary material, further inquiries can be directed to the corresponding author.

## Ethics statement

The studies involving humans were approved by Ethics Committee of Charles Nicolle Hospital in Tunis. The studies were conducted in accordance with the local legislation and institutional requirements. The participants provided their written informed consent to participate in this study.

## Author contributions

WZ and MK designed the study. WZ carried out the surgery. MK and GM carried out the measurements and follow-ups. MK, ZK, and SS wrote the manuscript. WZ and MK were involved in the analysis of the results. WZ and SS were involved in the revision of the manuscript. All authors contributed to the article and approved the submitted version.

## Conflict of interest

The authors declare that the research was conducted in the absence of any commercial or financial relationships that could be construed as a potential conflict of interest.

## Publisher’s note

All claims expressed in this article are solely those of the authors and do not necessarily represent those of their affiliated organizations, or those of the publisher, the editors and the reviewers. Any product that may be evaluated in this article, or claim that may be made by its manufacturer, is not guaranteed or endorsed by the publisher.
